# A new approach to study the sex differences in adipose tissue

**DOI:** 10.1186/s12929-018-0488-3

**Published:** 2018-12-03

**Authors:** Sarah Jayne Fitzgerald, Amol Vijay Janorkar, Allison Barnes, Rodrigo Oscar Maranon

**Affiliations:** 1 Department of Biomedical Materials Science, School of Dentistry, University of Mississippi Medical Center, Jackson, USA; 2 Department of Cell and Molecular Biology, University of Mississippi Medical Center, 2500 North State Street, Jackson, MS 39216 USA; 3 Department of Medicine/Nephrology, School of Medicine, University of Mississippi Medical Center, 2500 North State Street, Jackson, MS 39216 USA; 4 Mississippi Center for Excellence in Perinatal Research, University of Mississippi Medical Center, 2500 North State Street, Jackson, MS 39216 USA; 5 Cardio Renal Research Center, University of Mississippi Medical Center, 2500 North State Street, Jackson, MS 39216 USA; 6 The Women’s Health Research Center, University of Mississippi Medical Center, 2500 North State Street, Jackson, MS 39216 USA

**Keywords:** Adipocytes, Obesity, Sex differences, Stem cells

## Abstract

Obesity is one of the most invaliding and preventable diseases in the United States. Growing evidence suggests that there are sex differences in obesity in human and experimental animals. However, the specific mechanisms of this disease are unknown. Consequently, there is any particular treatment according to the sex/gender at this time. During the last decade, we observe a rise in the study of adipocyte and the possible mechanisms involved in the different roles of the fat. Furthermore, the effect of sex steroids on the adipocyte is one of the fields that need elucidation. Supporting evidence suggests that sex steroids play an essential role not only in the fat distribution, but also, in its metabolism, proliferation, and function. Thus, using in vitro and in vivo studies will contribute to our fight against this critical health public problem encompassing both sexes. In the present review, we discuss some of the recent advances in the adipocytes and the effect of the sex steroids on the adipose tissue. Also, we propose a new alternative to study the role of sex steroids on adipocyte biology through human adipose-derived stem cells.

## Introduction

Obesity is one of the most invaliding and preventable diseases in the United States (US). According to the National Health and Nutrition Examination Survey (NHANES – 2015 – 2016), the prevalence of obesity among adults aged 40–59 (42.8%) was higher than among adults aged 20–39 (35.7%) [1]. The panorama is discouraging since the problematic health is increasing without a possible solution in the meantime. The evidence is showing that in the United States, the obese incidence is growing at an alarming rate and it is expecting that by 2050 the prevalence of obesity in the adult population be approximately 42% [2].

Adipose tissue is accepted as a dynamic organ with a critical role in the physiology and pathophysiology of different diseases. In the past years, we observe an increased interest in the study of the adipose tissue and sex or gender related to the cardiometabolic disease. In this sense, studies in human and animals show that there is a sex difference in the susceptibility to develop obesity [3]. Between 2005 and 2014, the prevalence of overall obesity and extreme obesity increased significantly among women; however, there were no significant increases for men [4]. Women have a higher risk to get obesity than men of different age [5–7]. Although the evidence supports substantial differences in mechanisms and strategies of obese treatment in men and women, there is not a specific treatment according to the sex or gender [8].

In the present review, we discuss the adipocytes, its structure, function, and distribution, and the role of the sex hormones in the adipose tissue. Also, we propose a new alternative to study adipocyte biology through human adipose-derived stem cells.

## Adipocyte: Structure, function, and distribution

Obesity is the increase in body weight, but it is mainly at the expense of fat content. Adipose tissue is considered as an immune-metabolic organ, able to store energy and maintains the metabolic rate [9]. Mainly, adipocytes have different properties according to how the fat is composed. There are three partially accepted types of adipose tissue in the body which are structurally and functionally distinct: white adipose tissue (WAT: color give it to the lipid content), brown adipose tissue (BAT: color provided by the cytochromes in the mitochondria), and beige or bright adipose tissue (BeAT: color is a mix of white and brown adipose tissue).

**Fig. 1:**
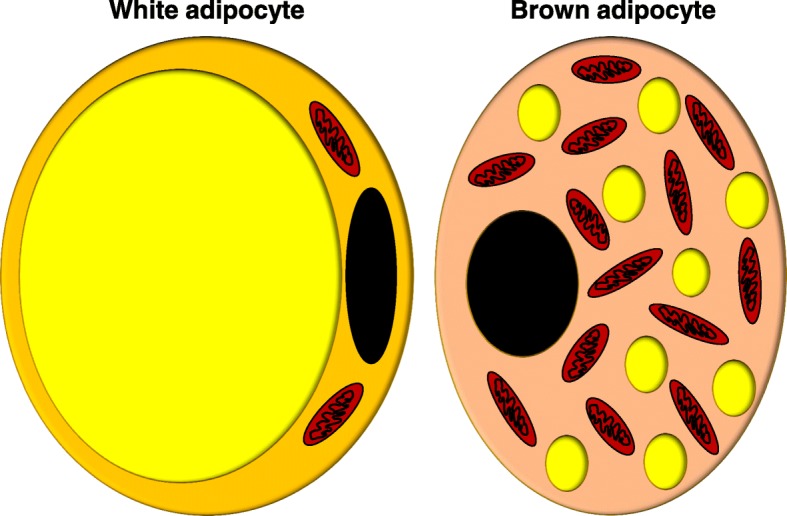
White and brown adipocytes. White adipocyte presents a significant droplet or inclusion of lipid into the cytoplasm who push the nucleus and organelles to the periphery. Brown adipocyte content multiple droplets of lipids with a high content of mitochondria

The WAT is characterized by a spherical shape that represents the mature adipocytes with a diameter size from 10 to 120 um [10]. They also present a significant droplet or inclusion of lipid into the cytoplasm who displaces the nucleus and organelles to the periphery (Fig. 1). Thus, the organelles and the flattened nucleus remain speared in a small space of the cytosol compressed between the lipid droplet and the basal membrane given the appearance of a signet ring [11, 12]. Numerous filaments of mitochondria are distributed in the perinuclear region.

WAT tends to form compartmental spaces which are a difference with the BAT [13]. The primary function suggested for WAT is lipid storage thanks to its ability to storage triglycerides in high levels with expansion during overfed. Also, this particular tissue contributes to the functioning of several systems in the body such as glucose homeostasis, inflammatory and immune response, and mechanical role [12]. However, its essential contribution is in the regulation of energy balance, where leptin plays a vital role. Leptin is a hormone secreted principally by the adipocytes and acts on peripheral targets and hypothalamic and extrahypothalamic centers controlling the nutritional state of the individuals [14–16]. WAT depot is localized in a different part of the body, but mainly in the abdomen region: the abdominal cavity (visceral fat) and under the skin (abdominal subcutaneous fat). This variety of fat is roughly associated with cardiovascular risk factors and contribute to insulin resistant and metabolic syndrome. It also associates with inflammatory cytokines, such as leptin, IL6, and TNFα [17, 18].

Another type of fat is the brown adipose tissue (BAT), which have structural and functioning differences with WAT. Its size is approximately among 15 to 50 um, and its form is ovoid. The primary function of BAT is thermoregulatory. However, BAT can play autocrine, paracrine, and endocrine roles [19]. Within of its autocrine role, BAT can secrete basement membrane protein such as collagen VI and fibronectin [20, 21]. Also, can synthesize and secrete adipsin (also known as Complement factor D), basic fibroblast growth factor, insulin-like growth factor I, prostaglandins, and adenosine [22, 23].

Regarding its paracrine function, BAT can secrete nerve growth factor, vascular endothelial growth factor, nitric oxide, and angiotensinogen [24, 25]. Finally, as an endocrine organ, BAT produces fatty acid, and only in special circumstances produces leptin, adiponectin, and resistin [26]. BAT was described as a protector factor from cardiovascular events, since produce cytokines that counterregulates the cytokines from the WAT stores. In human, BAT depot is localized mainly in the interscapulum and supraclavicular regions [27]. The subcutaneous BAT (scBAT) depot is larger than the BAT found in the thick neck embedded in the carotid sheath. Some individuals possess additional BAT depots in the axillary, prevertebral regions and kidneys, but scBAT is the BAT depot most consistently found in humans [28–34].

Recently was describe as a third type of fat, the beige or bright adipose tissue, that share almost all features of BAT except for the localization [10, 35]. It was found mixed with WAT in the visceral fat and is associated with the contribution to transform WAT into BAT. However, its function still needs further investigation.

According to the anatomical distribution, the fat can be called Visceral Adipose Tissue (VAT) or Subcutaneous Adipose Tissue (SAT) [27]. The VAT contains more adipocytes with characteristics of WAT, while the SAT include more adipocytes with attributes of the BAT. However, recent studies have shown that adipocytes from BAT could be mixed with adipocytes from WAT in the abdominal depot (beige adipose tissue) [36]. Table 1 summarizes the main features of the different types of fat.

**Table 1:** Main characteristics of white, brown, and beige adipocytes

	WHITE ADIPOSE TISSUE	Beige adipose tissue	brown adipose tissue
origin	- Myf5-negative cells	- Myf5-negative cells - White adipose tissue - Sca-1+ progenitor cells - Smooth muscle cells progenitors - PDGFRα+ cells	- Mainly Myf5-positive cells
LIPID DROPLET	Unilocular	Multilocular	Multilocular
NUCLEUS	Periferic	Middle	Middle
MITOCHONDRIA	Low	Low to High	High
function	Lipid/Energy storage	Thermogenesis (dependent on the stimuli)	Thermogenesis and Autocrine, Paracrine, Endocrine roles
human localization	Subcutaneous, intraabdominal	Cervical, parasternal, supraclavicular, para- and prevertebral	Interscapulum, supraclavicular, cervical, parasternal, para- and prevertebral, omental, axillary, kidneys
rodents localization	Inguinal subcutaneous, epididymal, Intraabdominal, epicardium	Subcutaneous axillar and inguinal, epicardium	Interscapulum, perivascular
cardiovascular disease	Positive related	Negative related	Negative related

It is necessary to highlight that the human adipose depots not always perfectly correlate in rodents (rats, mice). For example, the omentum contains a large percentage of visceral fat in humans, which is scarcely present in rodents. Conversely, the large epididymal fat pads of male mice, which are frequently sampled as representative of visceral fat, do not exist in men [37, 38].

## Adipose tissue: Sex steroids regulation in men and women

Obesity affects both sexes independent of the age, race, or culture. In general, females have more fat stores than men. Also, fat depots are distributed differentially in women and men, especially after the puberty because of the hormonal effect [39]. Before puberty, females have higher amounts of subcutaneous fat that age-matched boys. Women maintain this pattern, with more fat stores in the gluteal-femoral or peripheral regions which determine the typical pear form for women (Peripheral obesity or gynoid type). Some researchers associated this high subcutaneous fat stores in women with less cardiovascular risk compared to men [40]. Men tend to be more likely to have significant amounts of abdominal fat and to be more susceptible to abdominal adiposity (Central obesity or android type or apple form). Also, men are more likely to have higher visceral fat, but some paradoxical observation could be present if the fat stores increase hug amount in which obese men have more significant amounts of subcutaneous fat on their legs [41].

Sex steroids play an essential role in the production and function of fat, but also, in its metabolism, migration, and utilization. In women, estrogens play as a protector factor in preventing the fat accumulation particularly subcutaneous fat depots in postmenopausal women [42, 43]. Furthermore, through these actions, estrogens can reduce inflammatory signaling and improve insulin action [44].

The effect of estrogens through estrogen receptor α (ERα) is more related to WAT since the ovariectomy decreases its expression and adipocytes proprieties [44]. In ERαKO, mice without ERα in the whole body, there is an inhibition of the capacity of the adipocytes to store triglycerides affecting the adipocyte size and the total adiposity [44]. However, the mechanism by which estrogens exert this function is unclear.

Lazzarini and Wade indicated that estrogens could reduce the fat content by increasing the sympathetic nerve activity in the adipose tissue [45]. Nevertheless, it could be independent of ERs since there was no effect of fat denervation on fat pad weight or cytosol ER concentration in white adipose tissue in the animals treated with sesame oil. These data indicate that the sympathetic nerves may play a role in estrogen-induced reductions in fat pad weight but not via changes in adipose tissue cytosol estrogen receptors. Other studies suggested that estradiol could regulate the brown adipose tissue at the central level activating via hypothalamic AMPK [46]. However, the role of the ERs in the adipose tissue regulation needs further investigations.

On the other hand, androgens in premenopausal women have a deleterious effect, increasing the size of abdominal adipocytes and the wait/hip ratio; however, it does not affect the femoral adipocytes [47]. Also, androgens in this population was associated with increased plasma glucose and insulin levels, both basally and in response to oral glucose loading; and diminished in vivo insulin sensitivity, as revealed by increasing steady-state plasma glucose levels at comparable plasma insulin levels, attained by the infusion of somatostatin, insulin, and glucose [47]. Furthermore, in premenopausal female-to-male transsexual, Elbers et al. assessed fat depot by magnetic resonance imaging, and they showed that testosterone slightly increased the visceral fat area [48]. Contrary, in a study by Blouin et al. using omental and subcutaneous adipose tissue samples and adipose tissue explants, found that androgens, testosterone, and dihydrotestosterone, have an anti-adipogenic effect on the adipose tissue [49]. So, further studies are needed to a better comprehension of the role of androgen in the regulation of the adipose tissue in premenopausal women.

Clinical evidence on the literature agrees that androgens have a negative impact on the adiposity in women which can partially explain the higher cardiovascular and metabolic risk in women [50]. Recently, an elegant study by Michos and her group associates high testosterone levels alone with a higher risk of CVD and CHD in postmenopausal women [51]. Also, they reported that at this age, an elevated testosterone/estradiol ratio associate to women with a higher incidence of CVD, CHF, and HF [51]. In women with polycystic ovary syndrome (PCOS) and with high production of androgens also is associated with a high risk of insulin resistant and cardiovascular disease. The androgens in this particular case are produced by the ovaries and adrenal gland [52, 53]; however, interestingly, elevated levels of androgens are present in non-PCOS obese women. In this regards, Quinkler and colleagues have shown that adipose tissue can synthesize androgens [54], but if those androgens produced by adipose tissue are associated with a higher risk of CVD is unknown.

In males, androgens have the adipogenic capability which increases the insulin sensitivity and improves the insulin resistance. Also, androgens reduce the WAT/VAT content, body weight, and improves the metabolic syndrome [55]. This effect appears to be mediated by the androgen receptors (ARs) which have a high distribution in both visceral and subcutaneous fat [56].

There is a positive correlation between low levels of androgens (hypogonadism) and an increase of abdominal SAT and VAT in human and experimental animals [57, 58]. The testosterone supplements in those hypogonadic patients lead to a decrease in abdominal SAT and VAT [59]. However, new evidence of our laboratory and others showed that chronic treatment with testosterone increases the risk of hypertension and cardiovascular event. In our studies, we used two different strains of animals, the Zucker rats, an experimental obesity model, and the spontaneously hypertensive rats [60, 61]. Interestingly, although we observed in both strain a reduction in body weight and fat mass and improvement in some metabolic parameters with testosterone supplementation, it also induced an increase in blood pressure. In this sense, recently two clinical studies showed that testosterone supplementation increased the mortality rate attributable to cardiovascular disease [62, 63]. Furthermore, men who used anabolic steroids, a synthetic derivative of testosterone, had a higher risk of hypertension, ventricular remodeling, and sudden cardiac death [64]. This evidence suggests that testosterone supplementation act through different mechanisms that contribute to high blood pressure which is independent of upper body weight and fat mass. Also, it indicates that blood pressure needs to be carefully monitored during testosterone supplementation.

Today, although there is progress in the study of adipocytes biology and the sex steroids hormones as central regulators, one limitation in this field is the lack of an experimental model that could contribute to improving our understanding on the regulatory mechanisms that occur in different types of adipose tissue.

## Alternatives to study adipocytes biology

### The human adipose-derived stem cells (hASCs)

Stem cells have been used for several medical applications over the last few years, and in everything from basic science research to clinical applications. While the stem cells can come from several sources, the more common source is from a patient’s mesenchymal stem cells (MSCs) [65, 66]. The reasons for using the patient’s cells include the simplicity of obtaining these cells regarding availability, as well as to avoid the controversy surrounding embryonic stem cells. When the patient’s cells are used for clinical applications, there is a much lower risk of rejection or failure due to immune responses, and when used in basic or applied sciences, MSCs have excellent differentiation potential, providing an excellent source of study into the mechanics of stem cell differentiation. MSCs can be derived from several different sources, such as bone marrow, skin dermis, and adipose tissue, though some methods of collection are more advantageous than others [67]. Human adipose-derived stem cells (hASCs) has been a relatively new source for stem cells. They are abundantly found within adipose tissue, with approximately 1% of the tissue cells being stem cells in comparison to the meager 0.001–0.002% of mesenchymal stem cells (MSCs) found in bone marrow, and are easily procured during a minimally invasive surgery, including liposuction [65, 66]. Adipose tissue is also much less invasive and less painful procedure than the procedures used in obtaining the same amount of stem cells from bone marrow. Thus, the process for obtaining adipose tissue and the stem cells therein is much more feasible than obtaining the stem cells from the skin dermis or bone marrow. It is due to the size of the tissue, availability of the stem cells, and routine extraction of adipose tissue, making it overall the better option for retrieval by comparison. Because MSCs, and more specifically hASCs, have excellent differentiation potential, and they are readily available for isolation after any liposuction procedure provided informed consent, and IRB protocols are approved, stem cells derived from adipose tissue could be said to be the ideal and superior source for stem cell isolation and future stem cell research.

Unfortunately, hASCs have been shown to lose their ability to proliferate and to have decreased expression of pluripotent markers over time in vitro [68, 69]. It has been shown that they can maintain the expression of pluripotent markers, along with differentiation potential, for more extended periods of time if they are cultured under 3D culture format in vitro, instead of the conventional method of monolayer cultures [65]. Multicellular spheroids are widely used as a simple and effective 3D culture to mimic intracellular in vivo like conditions [70–72]. Later on, we describe the various methods available for stem culture for adipogenic differentiation.

## Advantages of new alternatives of three-dimensional cultures to study sex hormones in adipocytes

Traditionally, the characterization of adipocyte biology was performed on culturing pre-adipocytes using a two-dimensional (2D) cells culture model. Whereas such 2D approaches have been successful in elucidating the biology of subcutaneous adipocytes, these approaches have been suboptimal for recapitulating the biology of adipocytes from less robust sources such as visceral adipose tissue [73]. Also, planar 2D monolayer adipocytes cultures do not represent the complex architecture of adipose tissue in vivo, which is an advantage for the engineered three-dimensional (3D) adipose tissue model [74]. As a new alternative of in vitro model of adipose tissue, the 3D in vitro model of adipose tissue can be engineered as spheroids of adipocytes, which exhibit morphology similar to the native adipose tissue [75]_._ 3D adipocyte spheroids exhibit higher expression of adipogenic biomarkers, including triglycerides, peroxisome proliferator-activated receptor-γ (PPAR-γ), tumor necrosis factor α (TNFα), interleukin (IL)-1, IL- 6, and adiponectin, compared to the 2D planner model [74]. Recently, our group established a 3D spheroid model using human adipose-derived stem cells (hASCs) and their subsequent adipogenic differentiation [74].

Different studies have used isolated adipocytes to explain the role of sex steroids in adipogenesis which still is poorly understood. In isolated human adipocytes, 17 beta-estradiol can modulate the adipogenesis by increasing preadipocyte replication [76]. Also, high concentrations of dehydroepiandrosterone (DHEA) and other androgen-related steroids were shown to block the adipose conversion process, as followed by measurement of glycerol-3 phosphate dehydrogenase (GPDH) activity, a late marker of differentiation. On these studies, the authors used the 3 T3-L1 and 3 T3-F442A preadipocyte cell lines and pig preadipocytes respectively [77, 78]. Furthermore, Nishizawa et al. demonstrated that androgens could decrease adiponectin secretion in the 3 T3-L1 adipocytes. They suggested that in men, androgens could induce hypoadiponectinemia which may be associated with the insulin resistance and atherosclerosis in men [79]. Also, a growing body of evidence has shown that testosterone and DHT inhibit 3 T3 L1 adipocytes differentiation [79, 80]. However, to our knowledge, there is no evidence about the effect of androgen or estrogen using patient-derived hASC spheroids model.

## New alternatives to three-dimensional cultures

### Spheroid formation methods

#### a) 3D printed scaffolds

Bioprinting is used to include cells within a three-dimensionally printed scaffold as a means of forming cellular aggregates, making it essentially the biological equivalent of the computer-assisted design and manufacturing (CAD-CAM) systems. The general method is that a matrix containing cells, often a hydrogel, is extruded onto a surface in a preprogrammed pattern to form a 3D structure. Encapsulated cells then form spheroids within this hydrogel matrix, which can be as stiff or gel-like as required based upon the matrix and filler chosen in the design.

Type 1 collagen is most commonly used to act as the matrix for a printed hydrogel and can be printed in the form of a sphere to form “spheroids” in conjunction with an adipose-derived stromal vascular fraction (SVF) [81]. It is of this cell type, and the stem cells derived from this, we draw our focus. These particular spheroids were made using extrusion techniques onto superhydrophobic surfaces coated before printing. After polymerization, they were placed in a suspension bioreactor for incubation to allow the spheroids to mature into adipocytes [81]. Unfortunately, this method is highly sophisticated, and there are much simpler methods available to create a similar construct. The three-dimensional scaffold can also be achieved by using gelatin and sodium alginate as a matrix and filler. It is used in combination with a fibrinogen/cell mixture is used to create spheroids within the printed hydrogel, which is printed into a grid format to allow for oxygen and media to flow through freely ensuring that the spheroids receive greater nutrition in comparison to other scaffolds [81–84].

**Fig. 2:**
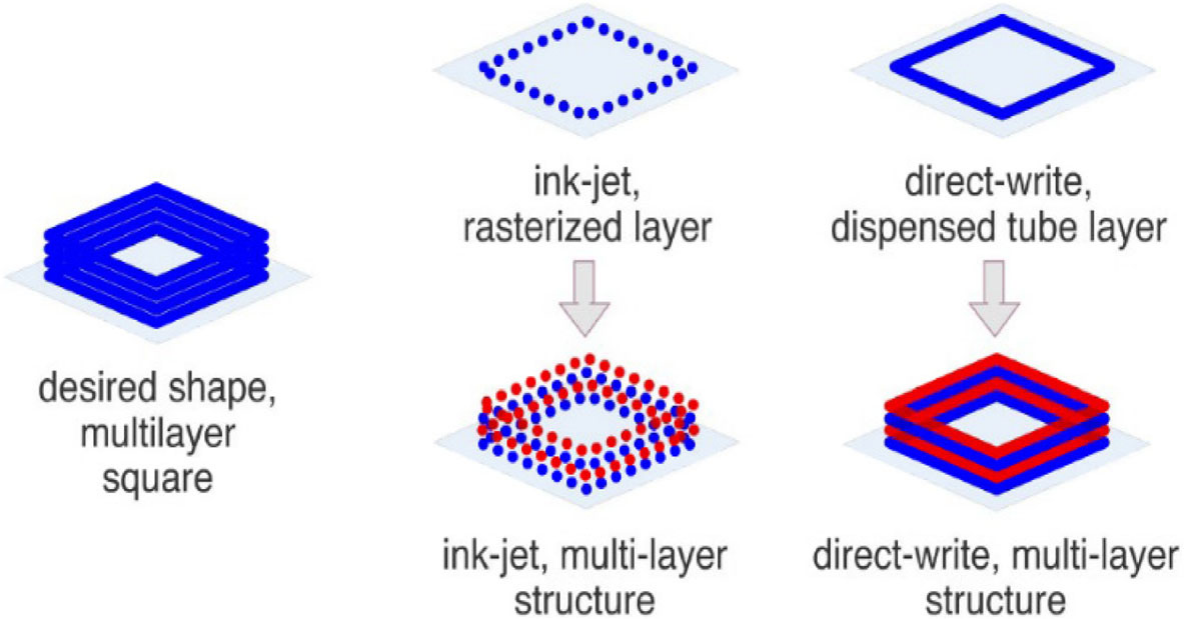
A schematic comparing the ink-jet and direct-write 3D printing methods. Ink-jet printing uses the “bottom-up” approach to create a three-dimensional scaffold and prints a “skeleton” of the object created by printing droplets of the material. Direct-write method prints the design layer by layer using pneumatic or mechanical power. (Reprinted with permission from [81])

The extrusion method is just as important as the extrusion matrix used. There are multiple extrusion techniques, including direct-write and ink-jet printing [81]. Ink-jet printing uses the “bottom-up” approach to create a three-dimensional scaffold as designed by a computer. This method prints a “skeleton” of the object created by printing droplets of the material through a custom-made needle of a commercial printer into the shape of the designed scaffold. Direct-write bioprinting, on the other hand, prints layer by layer the design created by the computer using a pneumatic or mechanical power through a syringe [81]. A schematic comparing the two are shown in Fig. 2. Both are used, though the direct-write method seems to be the one that is gaining popularity [82]. The direct-write method can also be used to print spherical shapes in the form of droplets within a solution. In one such instance, a cell/alginate mixture is added dropwise to a solution of calcium chloride, which forms the three-dimensional structure needed for these cells to form spheroids within the alginate gel [82]. This precision is useful, though one is unable to remove the cells for any quantitative assays that may be required.

#### b) Microfluidic devices

Microchips, also known as microfluidic devices, allow researchers to analyze cells by culturing them in restricted spaces, often only a few micrometers in size. Though there are many cell types that this method could be used for, our focus remains on stem cell proliferation and differentiation. The generalized way is that cells are forced into a three-dimensional formation by seeding them within a limited space and flowing media across them. This process allows for a three-dimensional culture on a micro scale and gives researchers control over the microenvironment surrounding the cells. Such culture systems can be directly purchased or can be custom printed based on a microfabricated template using a traditional 3D printer [85], and can be made by some biocompatible polymers, such as polydimethylsiloxane (PDMS). The use of these polymers has been variable in creating stable spheroids for long-term cultures, though their ability to provide a more in vivo like structure by having dynamic media flow has proven useful in cell-cell interactions studies [85].

**Fig. 3:**
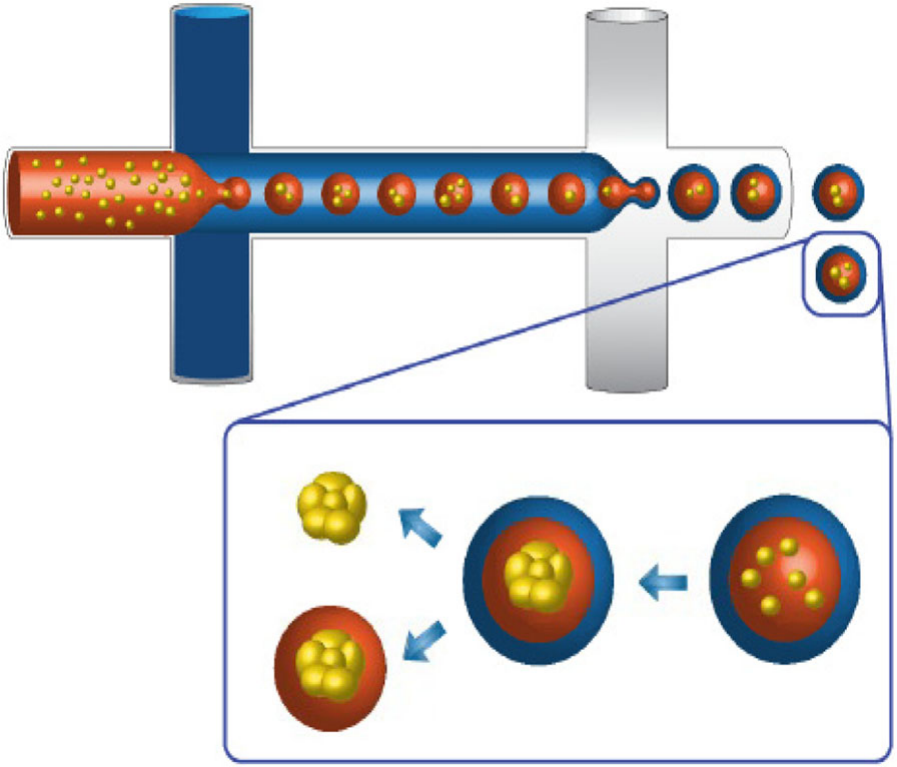
Schematic of the double-emulsion method of forming a microenvironment using microfluidic devices. The orange represents a water-based media, the blue represents an oil-based substance for first layer emulsion, and the white represents a water-based solution. (Reprinted with permission from [87])

In order to improve upon these devices, the culture conditions were altered rather than the materials. Some methods use micropatterning within their devices, while other ways use droplet-based microfluidic devices to create their own, controlled microenvironments, as shown in Fig. 3 [86–88]. This method uses droplets made of gel-based substances such as agarose, alginate, gelatin, or polyethylene glycol to encapsulate cells and allow them to form spheroids. These droplets can either be used within the microfluidic device or created using the device and act as a stand-alone device [85, 86]. Many reasons justify why one would want to culture cells in using microfluidic devices, including the small number of cells needed for each chip or condition, and the procedure can be conducted directly on a microscope for easy viewing. However, the cells cannot be removed from the microchip once they have been seeded.

#### c) Hanging drop

**Fig. 4:**
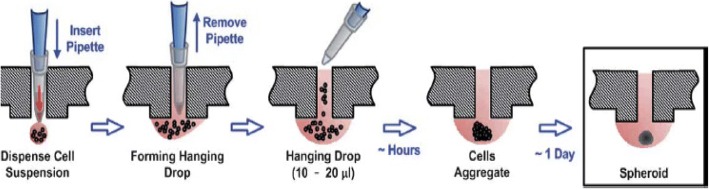
A cartoon depicting an example of cell seeding using the hanging drop method within the well of a specialized hanging drop plate that uses surface tension and gravity to form spheroids. The cells come together at the air-liquid interface and form a single spheroid. (Reprinted with permission from [91])

Hanging drop plates and trays can be bought from a manufacturer or created within the lab and exploits surface tension and gravity to form spheroids [89, 90]. The generalized method is that cells are seeded into a small amount of media within a micro-well plate. When inverted, the droplet creates an environment that allows for the cells to repel from the surface [89]. The cells come together at the air-liquid interface and form a single spheroid. The gas exchange here is relatively high, allowing the cells to maintain nutrition and oxygen for more extended periods than traditional cell cultures, though the resultant spheroids would need to be moved to another plate for long-term experiments, including those involving stem cell differentiation [91]. One main advantage of the hanging drop method is that it has a high throughput capability in that this method creates a uniform, reproducible spheroids, making them convenient for experiments with pharmaceutical applications [90, 91]. The fact that the spheroids take only a couple of days to form is advantageous. However, only one spheroid can be made within each well of a hanging drop plate/tray, thus requiring a more significant quantity of plates in comparison to other methods. There are also issues with the inversion process itself, though this is overcome by adding the cell suspension `through the access holes created in Fig. 4 [91].

#### d) Hydrogels

**Fig. 5:**
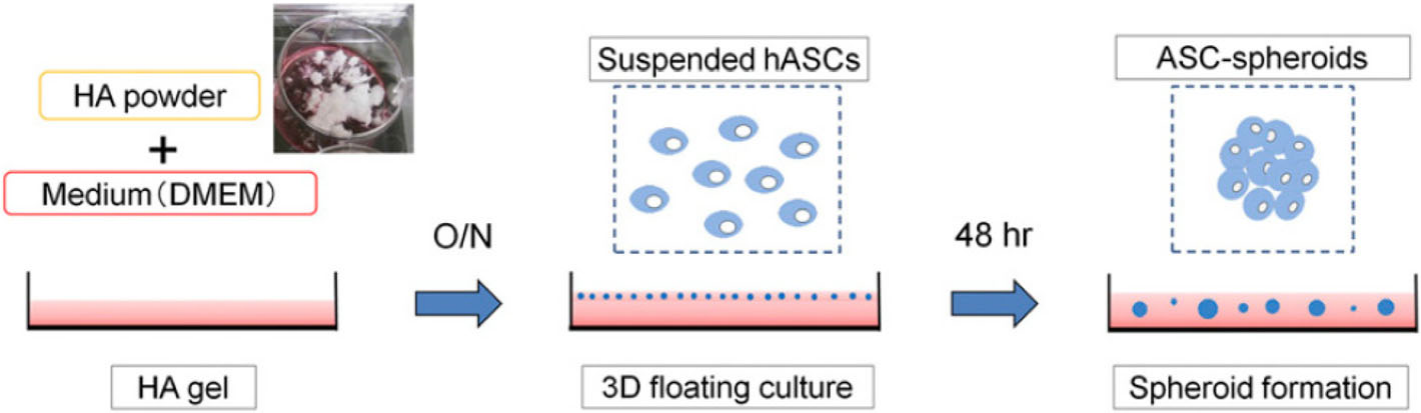
Schematic of the formation of spheroids within a hydrogel matrix. Cells are typically suspended in the precursor solution that sets to form the hydrogel. Mechanically stiff or chemically crosslinked hydrogels can “lock down” the encapsulated cells and lead to spheroid formation. (Reprinted with permission from [92])

Hydrogels are widely used as a three-dimensional cell culture model to develop spheroids (Fig. 5) [92]. The common gel-forming materials have been hyaluronic acid, polyethylene glycol, or different kinds of collagen, which could be obtained from human, rat, bovine, or goat [67, 92–95]. Optionally, additives can be incorporated as a way to “lock down” cells, provide therapeutic drug release, or to increase specific properties within the material. The intended change in property depends upon the additive, such as a crosslinker, which improves properties such as stiffness, modulus, and longevity [67, 92–95].

An important note to consider when using hydrogels in an experiment is the impact it may have on the cell type that is being used. This impact is especially significant in stem cell research, as it is not only important to consider the restraints placed upon the stem cells themselves, but also upon the differentiated lineage, the cells will follow. For example, osteogenic stem cell differentiation may require different mechanical properties within the hydrogel than stem cells differentiated as a chondrogenic lineage, just as they do in vivo [92]. Though hydrogels can be used for longer-term cultures, depending upon the materials that make up the hydrogel, another point to consider is if one can dissolve the hydrogel once the experiment is done without damaging the spheroids within the hydrogel. This fact also depends highly upon the components of the hydrogel itself and is usually digested by an enzyme such as collagenase, or diluted with media and separated by centrifugation [67, 92]. When cells are seeded within a hydrogel, especially one that is cross-linked, the ultimate proliferative capability of the cells will be less than in a monolayer, two dimensional culture, just because once the cells are “locked down” by the constraints of the hydrogel, they tend to form spheroids with the cells that are already available. This is especially true with hASCs, as their signal to stop proliferation is controlled by confluence [67, 92–95].

There are many advantages to using hydrogels for spheroid formation, in that the methods used in creating hydrogels is highly diverse and yet each is reasonably straightforward, and spheroid formation is highly reproducible. The type of cell, the cellular concentration at seed time, size of the spheroid intended, and removal of spheroids at end time are all points to consider in designing a hydrogel experiment.

#### e) Ultra-low attachment plates

Ultra-low attachment plates (ULA), have been used for many years and have helped expand the research of spheroid formation with their use on their own and as modified methods to create new ways of forming spheroids. The primary method is that the ULA plates are, at their surface, uninhabitable by cells [96]. Thus, the cells repel from the surface to form spheroids, creating a free-floating and three-dimensional culture within the media itself [67, 89]. The main attraction of this method is the simplicity of its design. However, the main drawback to using this method is to maintain the spheroids without losing them. When new media is exchanged for old, it is highly likely that some of the spheroids within the media will be aspirated with the older media and lost. This fact is highly disconcerting, as the more massive spheroids tend to be most easily moved by media exchange, thus losing the more physiologically relevant spheroids with each media exchange. One way to counteract this is to use the media itself as a way to gather data about the spheroids that were lost [96]. Media can contain the excretions of the cells within it, thus creating a kind of metric for how well the spheroids are doing based on their metabolic systems, which will be slightly different for each cell type. Amaral et al. used this method to determine the concentrations of lactate, glucose, and glutamine to track the cells’ metabolic activity [89]. Another way to accomplish this would be to centrifuge the aspirated media and re-suspend the separated spheroids with a small amount of fresh media and replaced into the well. However, this may interfere with the spheroid interaction and concentration within the well. This method, though highly straightforward, should consider the stem cell differentiation lineage when designing experiments with stem cells. When differentiating stem cells into an adipogenic lineage, for example, this method would not be recommended, only because adipocytes become more buoyant over time, and are more likely to be lost during media exchange.

#### f) Magnetic plates

Magnetic plates, or using magnetism to manipulate cells to form spheroids, is a relatively new method of three-dimensional cell culture. These plates are commercially available or can be made within the lab, by creating a hydrogel containing magnetic nanoparticles [97, 98]. One method employees a microfluidic device to generate gel beads, using oil/water emulsion, within each was a high concentration of cells and magnetic nanoparticles. These beads were then separated from the oil into a media buffer using a magnet to attract the cell beads, which were then transferred to a 96-well plate and cultured as normal [97]. Another method uses magnetic nanoparticles within a high cell concentrated well. When a magnet is passed beneath the wells, the cells are forced into a spheroid formation by the magnetic nanoparticles within the media [98]. Using magnetism to create a three-dimensional culture has many advantages, mainly because the method is incredibly easy to use. However, the cost of the nanoparticles in comparison to other methods may prove to be a hindrance for this method in the future.

#### g) Surface-tethered spheroid formation

**Fig. 6:**
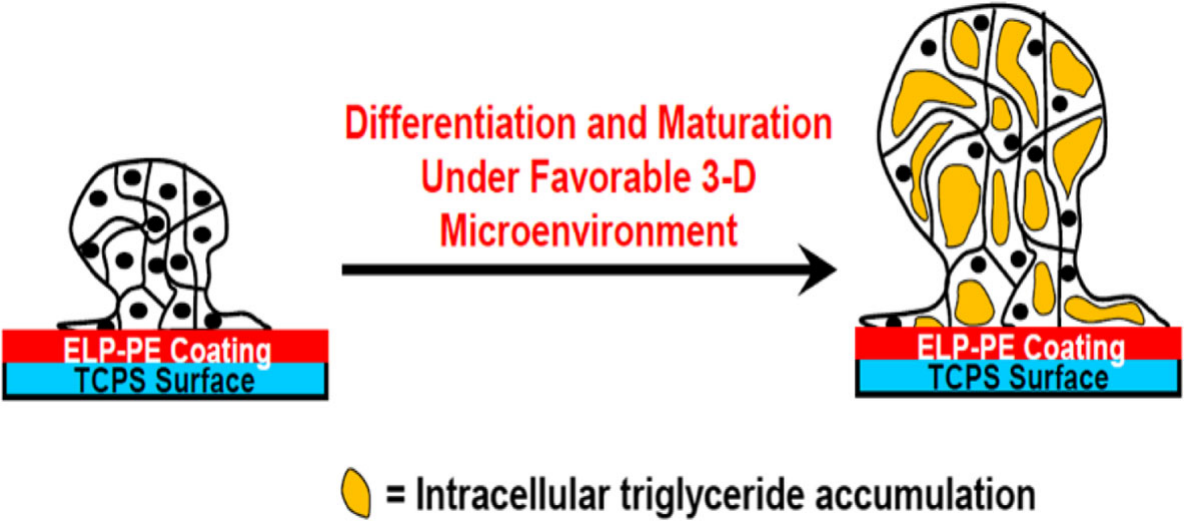
Schematic of differentiation of surface-tethered spheroids of hASCs atop a surface coated with elastin-like polypeptide-polyethyleneimine (ELP-PEI). The PEI repels the cells from the coated surface and induces spheroid formation, while the biocompatible ELP encourages the formed spheroids to adhere to the surface. Such surface-tethered spheroids can then be differentiated to the desired lineage (e.g., adipogenic lineage) by their differentiation and maturation under a suitable, physiologically-relevant microenvironment. (Reprinted with permission from [74])

Surface-tethered spheroids are created by changing the surface chemistry of the growth substrate. Elastin-like polypeptides (ELP) conjugated to polyelectrolytes (PE) have been used in coatings as a three-dimensional culture method for many different cell types. Here, the biocompatible ELP encourages cells to adhere to the surface, and the polyelectrolyte repels the cells from the coated surface. This process causes the cells to prefer one another to the surface, forming cell aggregates in the form of spheroids, while at the same time tethering to the surface (Fig. 6) [74]. The polyelectrolyte proven to be the most effective for these coatings within the past research is polyethyleneimine (PEI) [93, 99]. PEI has been shown to be cytotoxic on its own, but when conjugated to ELP, becomes biocompatible and induces spheroid formation. ELP-PEI coated surfaces are highly beneficial for culturing hASCs into the spheroid formation and subsequent differentiation of hASCs. The advantages of surface-tethered spheroids lie in the cells longevity, in that hASCs have been cultured using this method up to 21 days [99]. However, during their differentiation and maturation into adipocytes in vitro, stem cells differentiated along the adipogenic lineage begin to absorb fats during maturation and start to become more buoyant over time. Such fat-laden cells eventually lift off of the culture surface and are lost during the next media change. Because of the loss of physiologically relevant spheroids, elastin-like polypeptide-polyelectrolyte coated surfaces are used to form spheroids as a long-term culture method for mature adipocytes in vitro*,* in an attempt to tether the spheroids until the cell’s buoyant properties overcome the surface’s ability to keep the spheroid fastened to the surface [70–72, 93, 99].

## Conclusions

Adipose tissue is accepted as a dynamic organ with a critical role in the physiology and pathophysiology of different diseases. In this regards, the role of the sex hormones on adipocytes established sex differences in the distribution and regulation of adipose tissue in men and women. Both, androgen and estrogens hormones are present in both sexes; however, their specific role in the adipocytes need further investigations. In the current review, we discussed the recent advances of the adipocyte and the differences between white, brown, and beige adipose tissue. Also, we are proposing new alternatives for three-dimensional cultures to study the adipocytes biology and considering them as a possible tool to investigate the effect of sex hormones on the adipocyte. Finally, with a better understanding of the adipocyte biology and the role of the estrogens and androgen on them, we will be able to address sex-specific treatment alternatives for the obesity.

## Abbreviations

2D: Two-dimensional cells
3D: Three-dimensional cells
AMPK: 5′ adenosine monophosphate-activated protein kinase
ARs: Androgen receptors
BAT: Brown adipose tissue
BeAT: Beige adipose tissue
CAD-CAM: Computer-assisted design and manufacturing systems
CHD: Coronary heart disease
CHF: Congestive heart failure
CVD: Cardiovascular disease
DHEA: Dehydroepiandrosterone
ELP: Elastin-like polypeptides
ERs: Estrogen receptors
ERα: Estrogen receptor α
ERαKO: Mice without estrogen receptor alpha in the whole body
GPDH: Glycerol-3 phosphate dehydrogenase
hASCs: The human adipose-derived stem cells
HF: Heart failure
IL1: Interleukin 1
IL6: Interleukin 6
IRB: Institutional Review Board
MSCs: Mesenchymal stem cells
NHANES: National Health and Nutrition Examination Survey
PCOS: Polycystic ovary syndrome
PDMS: polydimethylsiloxane
PE: Polyelectrolytes
PEI: Polyethyleneimine
PPAR-γ: Peroxisome proliferator-activated receptor-γ
SAT: Subcutaneous adipose tissue
scBAT: Subcutaneous brown adipose tissue
SVF: Adipose-derived stromal vascular fraction
TNFα: Tumor Necrosis Factor alpha
ULA: Ultra-low attachment plates
VAT: Visceral adipose tissue
WAT: White adipose tissue
